# Subgroup detection in linear growth curve models with generalized linear mixed model (GLMM) trees

**DOI:** 10.3758/s13428-024-02389-1

**Published:** 2024-05-29

**Authors:** Marjolein Fokkema, Achim Zeileis

**Affiliations:** 1 Unit of Methodology and Statistics, Institute of Psychology, Leiden University, Leiden, The Netherlands; 2 Department of Statistics, Faculty of Economics and Statistics, Universität Innsbruck, Innsbruck, Austria

**Keywords:** Growth curve models, Decision trees, Model-based recursive partitioning, Mixed-effects models

## Abstract

Growth curve models are popular tools for studying the development of a response variable within subjects over time. Heterogeneity between subjects is common in such models, and researchers are typically interested in explaining or predicting this heterogeneity. We show how generalized linear mixed-effects model (GLMM) trees can be used to identify subgroups with different trajectories in linear growth curve models. Originally developed for clustered cross-sectional data, GLMM trees are extended here to longitudinal data. The resulting extended GLMM trees are directly applicable to growth curve models as an important special case. In simulated and real-world data, we assess performance of the extensions and compare against other partitioning methods for growth curve models. Extended GLMM trees perform more accurately than the original algorithm and LongCART, and similarly accurate compared to structural equation model (SEM) trees. In addition, GLMM trees allow for modeling both discrete and continuous time series, are less sensitive to (mis-)specification of the random-effects structure and are much faster to compute.

## Introduction

Development over time is of prime interest in psychological research. For example, in educational studies researchers may want to model student’s academic development over time; in clinical studies researchers may want to model patients’ symptoms over time. Mixed-effects or latent-variable models can be used to model such trajectories and allow for explaining heterogeneity with covariates of a-priori known relevance (e.g., McNeish & Matta, 2018). However, when these covariates or their shape of association with the response are not known in advance, methods for identifying them are needed.

**Fig. 1:**
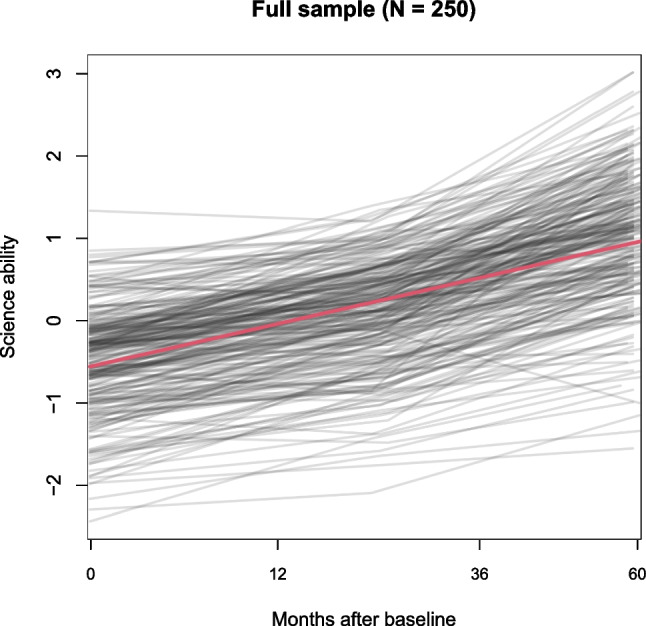
Growth curves of science ability. *Gray lines* depict observed individual trajectories, the *red line* depicts average growth curve as estimated with a linear mixed-effect model, comprising a fixed effect of time and a random intercept with respect to individuals. The *x*-axis is not linear in the number of months because time was scaled as $(\#\text {of months})^\frac{2}{3}$ in order to obtain approximately linear trajectories. To facilitate interpretation, the labels on the *x*-axes represent values on the original time scale

As an example, trajectories of science knowledge and skills from a sample of 250 children are depicted in Fig. 1. The children were assessed at three timepoints across grades 3 through 8.^1^ The red line depicts the estimated average trajectory, while the gray lines depict individual trajectories. The gray lines reveal substantial variability between the children, both in initial levels and growth over time. An obvious research aim would be to identify covariates that can explain or predict this heterogeneity.

### Recursive partitioning methods for growth curve models

**Fig. 2:**
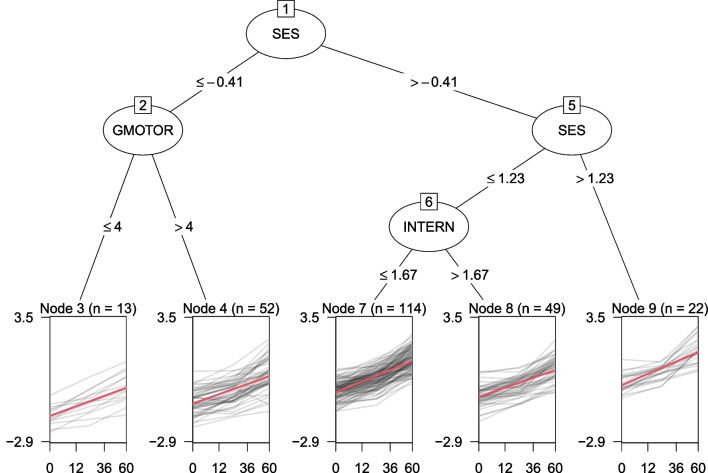
Partitioned growth curves of science ability. The *x*-axes represent the number of months after the baseline assessment, *y*-axes represent science ability. *Gray lines* depict observed individual trajectories. *Red lines* depict the average growth curve within each terminal node, as estimated with a linear mixed-effect model comprising node-specific fixed effects of time and a random intercept with respect to individuals. The *x*-axis is not linear in the number of months because time was scaled as $(\text {months after baseline})^\frac{2}{3}$ in order to obtain the approximately linear trajectories shown here. To facilitate interpretation, the labels on the *x*-axes represent values on the original time scale

Recursive partitioning methods (RPMs), also known as “trees“, allow for identifying relevant predictors from a potentially (very) large number of covariates. Figure 2 shows an example tree, which identified socio-economic status (SES), gross motor skills (GMOTOR), and internalizing problems (INTERN) from a set of 11 socio-demographic and behavioral characteristics of the children, assessed at baseline. Five subgroups were identified, corresponding to the terminal nodes of the tree, each with a different estimate of the fixed intercept and slope, which are presented in Table 1. Groups of children with higher SES also have higher intercepts, indicating higher average science ability. The group of children with lower SES (node 2) is further split based on gross motor skills, with higher motor skills resulting in a higher intercept. The group of children with intermediate levels of SES (node 6) is further split based on internalizing problems, with lower internalizing problems resulting in a higher intercept. The two groups (or nodes) with higher intercepts also have higher slopes, indicating that children with higher baseline ability may also gain more ability over time.

Because trees are non-parametric methods, they do not directly provide valid estimates of uncertainty. Athey & Imbens (2016) propose a sample-splitting procedure, where one sample is used for split selection and a separate sample is used to compute node statistics (e.g., means and variances). The right part of Table 1 presents node-specific intercepts, slopes and standard errors computed on a new sample of 250 children. Naturally, the coefficients computed on the new sample differ somewhat from those computed on training data, but the ranking of node-specific intercepts and slopes is very similar between the two samples.

**Table 1:** Estimated fixed-effects coefficients for the terminal nodes of Fig. 2

	Training data			Test data				
Node	Intercept	Slope	*n*	Intercept	s.e.	Slope	s.e.	*n*
3	$-1.576$	0.094	39	−0.966	0.155	0.089	0.008	45
4	$-0.942$	0.092	156	−0.860	0.078	0.091	0.004	177
7	$-0.350$	0.104	342	−0.421	0.052	0.108	0.003	309
8	$-0.595$	0.090	147	−0.649	0.080	0.096	0.005	135
9	0.009	0.111	66	−0.161	0.119	0.103	0.006	84

*Note*. Training data refers to the observations also depicted in Figs. 1 and 2, test data refers to a new sample, used to (re)compute node-specific statistics and obtain valid uncertainty estimates. s.e. = standard error, *n* = node-specific sample size

The tree in Fig. 2 has been estimated with generalized linear mixed-effects model trees (GLMM trees; Fokkema et al., 2018). GLMM trees were originally proposed for subgroup detection in clustered cross-sectional studies, where subjects are nested in treatment centers, classrooms and/or geographical areas, for example. In the current paper, we extend GLMM trees, so that they can be applied to partitioning linear growth curve models (LGCMs). The general idea of GLMM trees is appealing for subgroup detection in almost any type of mixed-effects model. Compared to clustered cross-sectional data, however, longitudinal data may require a different estimation approach: The variance of random effects tends to be higher with longitudinal data, and the predictors of interest tend to be measured at higher levels (e.g., time-invariant covariates). In this paper, we propose and test two extensions of GLMM trees that account for these characteristics. We focus on the specific use case of partitioning LGCMs, but the extensions are critical for a wider range of settings where covariates are measured at higher levels and/or where the random effects have substantial variance.

There are several alternative RPMs that can be used to partition LGCMs: GUIDE (Loh, 2002), longRPart (Abdolell et al., 2002), GEE-based decision trees (Lee, 2005), longitudinal interaction trees (IT; Su et al., 2011), SEM trees (Brandmaier et al., 2013), mixed-effects longitudinal trees (MELT; Eo & Cho, 2014) and LongCART Kundu & Harezlak (2019). Further, the longRPart2 (Stegmann et al., 2018) and IT-LT (Wei et al., 2020) methods allow for subgroup detection in non-linear growth curve models.^2^

The main characteristic that sets GLMM trees apart from other methods for partitioning LGCMs is a local-global estimation approach: GLMM trees do not fit a full parametric model in each of the subgroups defined by the terminal nodes of the tree. Instead, fixed-effects parameters are estimated *locally*, using the observations within a terminal node, while the random-effects parameters are estimated *globally*, using all observations. This local-global estimation approach was first proposed by Hajjem et al. (2011) and Sela & Simonoff (2012) for trees with constant fits (i.e., intercepts only) in the terminal nodes. With GLMM trees, the approach was generalized to allow for GLMs with any number of parameters in the terminal nodes, thus allowing for non-Gaussian responses and targeted detection of a wide range of possible interaction effects in mixed-effects models (Fokkema et al., 2018).

Other methods for partitioning LGCMs take a fully local estimation approach: Within every node or subgroup defined by the terminal nodes, a full parametric model is estimated based on the observations in that subgroup only. This fully local estimation approach provides more flexibility, but also higher computational burden and model complexity. In contrast, GLMM trees estimate a (much) lower number of random-effects parameters, which likely reduces overfitting and improves stability and generalizability of the results. Furthermore, the fully local estimation requires possible partitioning variables to be measured at the highest level of nesting, while GLMM trees’ local-global estimation approach allows partitioning variables to be measured at any level.

The computational advantage of GLMM trees is strongest compared to longRPart, longRPart2, IT-LT and LRT-based SEM trees. These methods employ an exhaustive split detection procedure, where for every possible split point in the current node, the full parametric model needs to be re-estimated in the two resulting nodes. To choose the optimal split, the splitting criterion (such as a *p*-value from a likelihood-ratio test) is derived from these two models. Not only does this bring a heavy computational load, but it also introduces a selection bias towards covariates with a larger number of possible cutpoints (Shih & Tsai, 2004; Shih, 2004). LongCART, MELT, GEE-based decision trees and score-based SEM trees also fit full parametric models in each of the nodes, but do not require model refitting for cutpoint selection; they employ the predictions or residuals from the fitted model in the current node for selecting the best split. This reduces computational load, while it also allows for separating variable and cutpoint selection, thus preventing selection bias. The GLMM tree algorithm shares these advantages, because it also employs a two-step approach to split selection.

Given their unbiased variable selection, lower model complexity and computational burden, GLMM trees might be particularly useful for subgroup detection in LGCMs. The next section explains how GLMM trees are estimated and propose adjustments for partitioning longitudinal trajectories. Next, the performance of the proposed adjustments is evaluated: In Study I, we assess performance in simulated datasets, in Study II, we compare performance of GLMM trees with that of two other partitioning methods: SEM trees and LongCART. In Study III, we assess performance of the proposed adjustments in real datasets on children’s development of reading, math and science abilities. In the Discussion, we summarize our findings and discuss implications.

## Estimation of GLMM trees and adaptations for longitudinal data

In the GLMM tree model (Fokkema et al., 2018), expectation $\mu _i$ of outcome vector $y_i$ is modeled through a linear predictor and suitable link function:

1 $$\begin{aligned} E[y_i | X_i]= &  \mu _i, \end{aligned}$$

2 $$\begin{aligned} g(\mu _{i})= &  X_{i} \beta _{j} + Z_{i} b_{i} \end{aligned}$$

Throughout this paper, we focus on the case with a continuous, normally distributed response $y_i$ with constant variance $\sigma _\epsilon ^2$. Therefore, the identity function can be used for the link *g*, but generalizations to other response variable types within the GLM are straightforward. In the general notation above, $X_i$ is the $n_i \times (p + 1)$ fixed-effects design matrix for subject *i* $(i = 1, \dots , N)$, comprising *p* time-varying covariates plus one column of 1s for the intercept. In this paper, we assume that time is the only time-varying covariate of interest (i.e., $p = 1$), but other time-varying covariates can also be included in $X_i$. The number $n_i$ and spacing of observed timepoints may differ between subjects. Time-constant covariates are not contained in $X_i$, but enter the model through the subgroup indicator *j*. The value of the fixed-effects parameters $\beta$ (here, intercept and time slope) in GLMM trees are node-specific, their value depending on the subgroup/node *j* into which subject *i* falls. In the current paper, subgroup membership is defined by the values of time-constant covariates.

As in the standard GLMM (e.g., Pinheiro & Bates, 2000) $Z_i$ is the random-effects design matrix for subject *i*, comprising a subset of columns of $X_i$, and $b_i$ is the corresponding vector of random effects for subject *i*. Finally, $b_i$ is assumed to follow a (possibly multivariate) normal distribution with mean zero and (co)variance $\Sigma$.

The parameters of a traditional GLMM can be estimated, among other techniques, by maximum likelihood (ML) or restricted ML (REML). Thus, when it is known into which node *j* each subject *i* falls, the GLMM specified by Eq. 2 can be fitted “as usual”, yielding *local* subgroup-specific fixed-effect estimates $\hat{\beta }_j$ and *global* random-effect estimates $\hat{b}_i$. To infer the subgroup membership for all observations *i*, the random-effect estimate is treated as a known offset and a GLM tree is estimated using the model-based (MOB) recursive partitioning algorithm of Zeileis et al. (2008). The overall GLMM tree model is then estimated by alternating between estimating the partition (i.e., subgroups or terminal nodes *j*), and estimating the random- and fixed-effects parameters, as per the following algorithm:

0.: Initialize by setting step $r = 0$ and all random-effect estimates $\hat{b}_{i,(r)} = 0$.
1.: Set $r = r+1$. Fit a GLM tree using $Z_{i} \hat{b}_{i,(r-1)}$ as an offset, yielding the partition $j_{(r)}$.
2.: Fit the mixed-effects model $g(\mu _{i}) = X_{i} \beta _{j, (r)} + Z_{i} b_{i, (r)}$ with the partition $j_{(r)}$ from Step 1. Extract the random-effect estimates $\hat{b}_{i,(r)}$ from the fitted model.
3.: Repeat Steps 1 and 2 until convergence.

This initialization simply assumes zero random effects. Convergence of the algorithm is monitored through the log-likelihood of the mixed-effects model fitted in Step 2. Typically, this converges when the partition $j_{(r)}$ from the GLM tree in Step 1 is the same as $j_{(r-1)}$ from the previous iteration.

The following two subsections describe alternative approaches for the initialization in Step 0 and for fitting the GLM tree in Step 1. Each subsection first reviews the well-established methods and then proceeds to discuss modifications that may improve performance when partitioning longitudinal data.

**Fig. 3:**
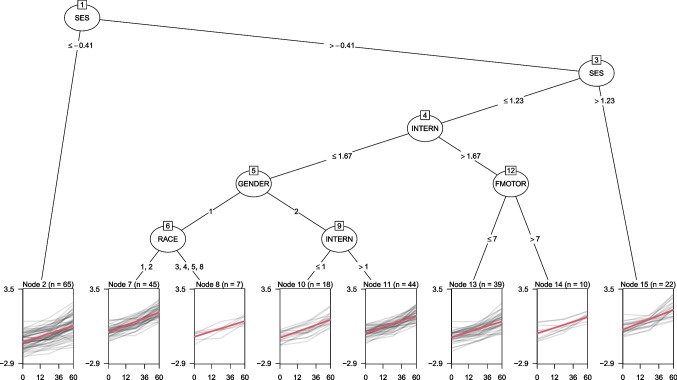
GLMM tree estimated by initializing with zero random effects. *Note*. The *x*-axes represent the number of months after the baseline assessment, the *y*-axes represent science ability. *Gray lines* depict observed individual trajectories. *Red lines* depict the average growth curve within each terminal node, as estimated with a linear mixed-effect model with a node-specific fixed effect of time and random intercepts estimated with respect to individuals

### Initialization

Previous studies on mixed-effects recursive partitioning find that initializing the random-effect estimates with zero yields accurate estimates of subgroup memberships and final models (Hajjem et al., 2011; Hajjem et al., 2014; Hajjem et al., 2017; Sela & Simonoff, 2012; Fu & Simonoff, 2015; Fokkema et al., 2018). Sela & Simonoff (2012) assessed the impact of different initialization values and found only minor differences that decreased with increasing sample size. In Fokkema et al. (2018), we found initializing estimation of GLMM trees with zero random effects performed well in cross-sectional clustered data. With longitudinal data, however, random effects tend to be more pronounced: Repeated measures on the same subjects tend to be correlated more strongly than observations nested within the same unit in cross-sectional data. If random effects are sizable, the initial assumption of zero random effects could provide an unrealistic starting point that may be difficult to overcome in subsequent iterations. We expect that for partitioning LGCMs, initializing estimation with the random effects instead of the subgroup structure may improve subgroup recovery. Specifically, that means the GLMM-tree algorithm starts by estimating the classic version of the mixed-effects model from Eq. 2 with just one set of fixed-effects coefficients $\beta$ and all subjects in a single group.

The alternative initialization step is thus:

Initialize by setting step $r = 0$ and fit the mixed-effects model $g(\mu _{i}) = X_{i} \beta + Z_{i} b_{i, (r)}$ to the full sample. Extract the random-effect estimates $\hat{b}_{i,(r)}$ from the fitted model.

To illustrate, we applied both initialization approaches to the dataset from Fig. 1. The tree in Fig. 2 was in fact estimated by initializing with the random effects. Initializing estimation assuming zero random effects resulted in the tree in Fig. 3. The split based on gross motor skills was not implemented, while additional splits were implemented based on gender, race, internalizing problems and fine motor skills. In light of the small number of respondents for some of the subgroups in Fig. 3, these effects may not generalize well to other samples. Therefore, generalizability of the tree structures obtained will be empirically evaluated using cross validation in Study III.

Table 2 presents the estimated random-effects parameters for the models fitted thus far. Compared to the global LMM in Fig. 1, the trees in Figs. 2 and 3 have very similar residual variances, but clearly lower variance of the random intercept. Part of the inter-individual variation captured by the random effects in the global LMM is thus explained by subgroup-specific fixed effects in the trees.

**Table 2:** Estimated random-effects parameters for the mixed-effects models in Figs. 1 through 4

Figure	Model	Estimation	*J*	$\hat{\sigma }_{b}$	$\hat{\sigma }_{\epsilon }$
1	LMM	default	1	0.610	0.349
2	LMM tree	random effects initialization	5	0.459	0.346
3	LMM tree	default	8	0.452	0.346
4	LMM tree	clustered covariances	4	0.475	0.346

Note. *J* is the number of subgroups; $\hat{\sigma }_b$ is the estimated standard deviation of the random intercept; $\hat{\sigma }_\epsilon$ is the estimated residual standard deviation

### Partitioning

The subgroup structure in Step 1 of the GLMM-tree algorithm is estimated by a GLM tree using the model-based recursive partitioning (MOB) algorithm of Zeileis et al. (2008). Here, we give a general overview and then comment on aspects of the algorithm particularly relevant for partitioning LGCMs. In the case of GLMs (with random effects held constant in an offset for GLMMs), the MOB algorithm cycles iteratively through the following steps:

- Fit the GLM to all observations in the current subgroup.
- Test for instability of the GLM parameters with respect to each of the partitioning variables.
- If there is some overall parameter instability, split the subgroup with respect to the partitioning variable associated with the highest instability.
- Repeat Steps (a) through (c) in each of the resulting subgroups.

Parameter stability in Step (b) is tested using the *scores* (gradient contributions) from the GLM fitted in Step (a). Under correct specification of the model and mild regularity conditions, the scores have an expected value of 0. The parameter stability tests evaluate whether the scores fluctuate randomly around this mean of 0, or exhibit systematic deviations when ordered by the values of a partitioning variable. For continuous covariates $u_k$ (or ordered covariates with a large enough number of unique values), this involves computing the following cumulative score process $W_k(t)$ with respect to each of the potential partitioning variables (Zeileis et al., 2008):

3 $$\begin{aligned} W_{k}(t) = \hat{J}^{-1/2} n_{j}^{-1/2} \sum ^{[n_jt]}_{i=1}{\hat{\psi }}_{\sigma (u_{ik})} \end{aligned}$$

where $\hat{J}$ is a suitable estimate of the covariance matrix of the parameter estimates, and $n_j$ gives the number of observations in the current subgroup. Further, $\hat{\psi }_{\sigma (u_{ik})}$ denotes the scores evaluated at the parameter estimates, with subscript $\sigma (u_{ik})$ denoting their ordering by the values of partitioning covariate $u_k$. Note that $0 \le t \le 1$, thus $n_jt = 1$ for an observation associated with a unique minimum on the partitioning variable, and $n_jt = n_j$ for an observation with a unique maximum.

From the cumulative score process $W_k(t)$, a range of test statistics can be derived which capture increased fluctuations (beyond the random fluctuation under parameter stability). For numerical partitioning variables, a maximum Lagrange multiplier test statistic can be computed, which takes the maximum of the squared Euclidean norm of $W_k(t)$, weighted by its variance (Zeileis & Hornik, 2007). This statistic is referred to as the *supLM* statistic, and is asymptotically equivalent to the maximum of likelihood-ratio statistics. Approximate asymptotic *p* values for the *supLM* statistic can be computed with the method of Hansen (1997). Categorical covariates do not provide an implicit ordering and scores are therefore binned at each level of the covariate. From these, a test statistic is computed that does not depend on the ordering of the levels (Merkle et al., 2014).

**Fig. 4:**
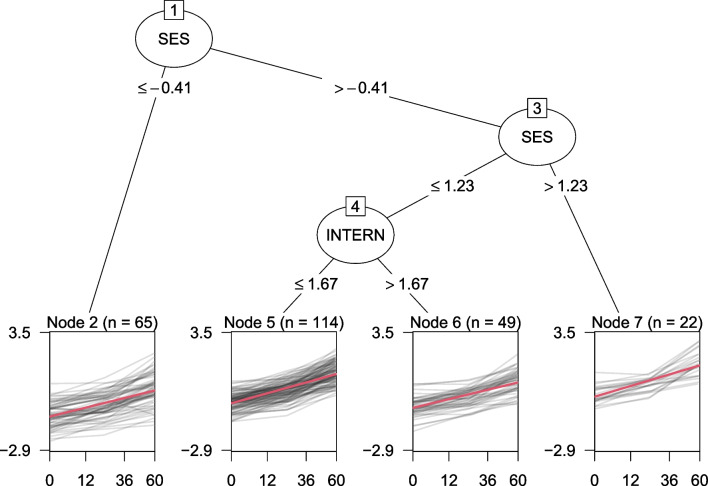
GLMM tree estimated with cluster-level parameter stability tests. The *x*-axes represent the number of months after the baseline assessment, *y*-axes represent science ability. *Gray lines* depict observed individual trajectories. *Red lines* depict the average growth curve within each terminal node, as estimated with a linear mixed-effect model with a node-specific fixed effect of time and random intercepts estimated with respect to individuals

When partitioning longitudinal data, covariates will often be measured at the subject level (i.e., time-invariant covariates), which should be accounted for in computing the estimated covariance matrix $\hat{J}$. In general, this computation makes use of the scores. By summing the scores within clusters prior to computation of the covariances, so-called *clustered* covariances are obtained, which account for dependence between observations within the same cluster (Zeileis et al., 2020). This resembles a GEE-type approach with an independence correlation structure. We expect that in partitioning LGCMs, use of clustered covariances in the parameter stability tests will improve subgroup recovery.

To illustrate, the tree in Fig. 4 was estimated using cluster-level parameter stability tests. Compared to the trees in Figs. 2 and 3, this yielded the most parsimonious tree structure thus far. The random-effects parameters in Table 2 show that this smaller GLMM tree also has slightly higher variance of the random intercept. With less subgroups or terminal nodes, less variance can be captured by the tree structure, and more variance can be captured by the random effects. Fitting a GLMM tree with both cluster-level parameter stability tests and random-effects initialization resulted in the exact same tree structure and parameter estimates. In the following sections, we will evaluate whether cluster-level parameter stability tests and/or random-effects initialization provide more accurate and better generalizable GLMM trees.

**Fig. 5:**
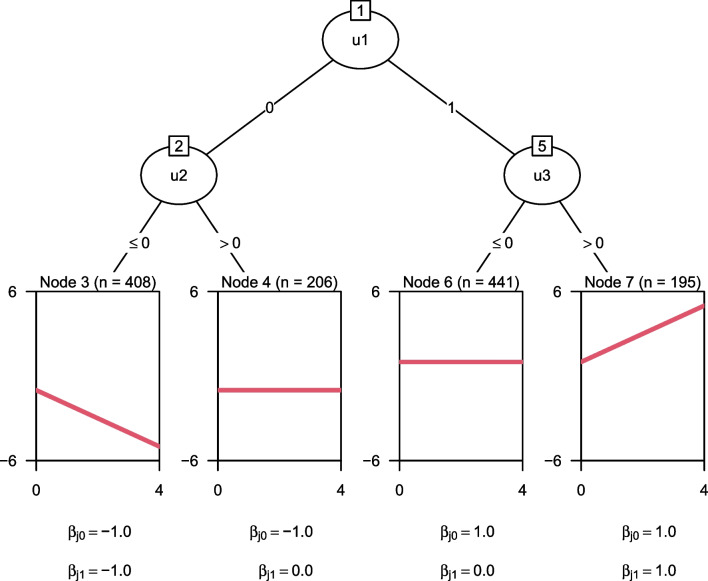
Design of subgroups and fixed effects

## Study I: Assessment of subgroup recovery

### Method

#### Data generation

We simulated data according to the subgroup structure depicted in Fig. 5. Every dataset comprised four non-overlapping subgroups, corresponding to the terminal nodes of Fig. 5, defined by the three true partitioning variables: $u_{1}, u_{2}$ and $u_{3}$. All partitioning variables were generated from a standard normal distribution with $\mu = 0$ and $\sigma ^2 = 25$. To allow for assessing possible selection bias toward partitioning variables with a larger number of possible cutpoints, variable $u_1$ was transformed to a binary factor, with values below the mean set to 0 and values above the mean set to 1. The response was computed as:

$$\begin{aligned} y_{i} = X_{i} \beta _{j} + Z_{i} b_i + \epsilon _{i}, \end{aligned}$$

where $\beta _j$ corresponds to the fixed effects in terminal node *j* of which subject *i* is part. $\beta _j$ values are reported below the terminal node panels in Fig. 5. The fixed- and random-effects design matrices $X_i$ and $Z_i$ are identical, each comprising two columns: a vector of 1s for the intercept, and a vector of timepoints. The same set of timepoints was generated for all subjects: 0, 1, 2, 3, 4.^3^ Values of $b_i$ (random intercepts and slopes) were generated from a multivariate normal distribution with mean zero and a $2 \times 2$ diagonal covariance matrix $\Sigma$, the diagonal entries determined by the level of the data-generating design described below. Values of $\epsilon _{i}$ were independently generated from a normal distribution with $\mu = 0$ and $\sigma ^2 = 5$.

We varied the following five data-generating characteristics:

1. Number of subjects: small ($N = 100$) or large ($N = 250$).
2. Variance of the random intercept: small ($\sigma _{b_0}^2 = 1$) or large ($\sigma _{b_0}^2 = 4$).
3. Variance of the random slope: small ($\sigma _{b_1}^2 = 0.1$) or large ($\sigma _{b_1}^2 = 0.4$).
4. Number of noise variables: small ($p = 5$) or large ($p = 25$).
5. Intercorrelation between partitioning variables: absent ($\rho = 0$) or present ($\rho = 0.3$).

Expressed as effect sizes, the values chosen for $\sigma _{b_0}$ correspond to intra-class correlations of 0.167 and 0.444, respectively. Values chosen for $\sigma _{b_1}$ correspond to effective curve reliabilities of 0.219 and 0.286 for $\sigma _{b_0}^2 = 1$, and 0.528 and 0.615 for $\sigma _{b_1}^2 = 4$, as computed per Brandmaier et al. (2018). A full factorial design was employed, yielding $2^5 = 32$ cells of the design; 100 repetitions were performed per cell. All data generation and analysis was performed in R (version 4.1.2; R Core Team, 2022).

#### Model fitting

We applied ten different fitting approaches to every generated dataset. Each variation combines one of three *random-effects specifications* (none vs. intercepts vs. intercepts+slopes) with one of two *random-effect initializations* (if any; all zero vs. full sample estimates) and one of two *covariance specifications in the parameter instability tests* (classic vs. clustered):

- None: $\hat{\sigma }_{b_0} = \hat{\sigma }_{b_1} = 0$. Random effects are fixed to 0, yielding linear model (LM) trees with fixed effects only and the following variations of covariance specifications:
  - Default: Classic observation-level covariances.
  - Alternative: Clustered covariances.
- Intercepts: $\hat{\sigma }_{b_0} > 0; \hat{\sigma }_{b_1} = 0$. This yields linear mixed-effects model (LMM) trees in which the variance of the random intercept was freely estimated and the variance of the random slope was fixed to 0. The four variations considered are the following:
  - Default: Classic observation-level covariances in the parameter stability tests and random-effect initialization with all zeros (original Step 0).
  - Alternative: Clustered covariances in the parameter stability tests.
  - Alternative: Random-effect initialization with the full-sample estimates (alternative Step 0$'$).
  - Alternative: Clustered covariances and random-effect initialization with the full-sample estimates.
- Intercepts and slopes: $\hat{\sigma }_{b_0} \!>\! 0; \hat{\sigma }_{b_1} \!>\! 0$. This yields LMM trees in which the variance of both random intercept and slope were freely estimated. The four variations considered are the same as for the intercept-only LMM trees.

**Fig. 6:**
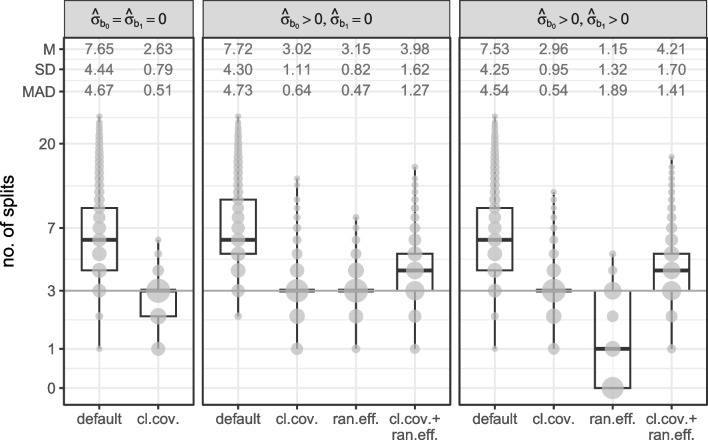
Tree size distributions for LM trees (*left panel*) and LMM trees (*middle* and *right panel*). M = mean number of splits; SD = standard deviation of the number of splits; MAD = mean absolute deviation from true tree size. *Gray circles* represent counts, *dark gray horizontal lines* represent true number of splits 3. Panel labels indicate whether variances of the random intercept and slope were fixed to 0 or freely estimated. Distances on *y*-axis are on the log scale. cl.cov. = cluster-level covariances employed in parameter stability tests; ran.eff. = estimation initialized with the random effects on the full sample

To fit LM trees, we used package *partykit* (version 1.2-15; Hothorn & Zeileis, 2015). To fit LMMs, we used package *lme4* (version 1.1-29; Bates et al., 2015). To fit LMM trees we used package *glmertree* (version 0.2-0; Fokkema et al., 2018). To compute clustered covariances, we used package *sandwich* (version 3.0-1; Zeileis et al., 2020). We employed the outer product of gradients method to compute covariances, thus employing only the meat of the sandwich estimator.

#### Evaluation of performance

We evaluated tree accuracy by counting the number of splits in every tree, and computing the standard (SD) and mean absolute deviation (MAD) from the true tree size of three splits. A tree with more than three splits was thus taken as type I error, while a tree with less than three splits was taken as a type II error (i.e., power too low to detect the true partitioning variables). We also assessed whether the variable selected for the first split in every tree was the true first splitting variable.

## Results

Figure 6 depicts the number of splits implemented by each partitioning approach. The default fitting approach tends to overfit, irrespective of the random-effects specification, implementing $> 3$ splits in 89% datasets, on average. The use of cluster-level covariances in the parameter stability tests successfully mitigated overfitting: LMM trees with clustered covariances with (middle panel) or without (right panel) random slopes showed an average number of splits closest to the true tree size.

In terms of MAD, however, LMM trees initializing estimation with the random effects performed best, but only if the random slope was not estimated. Thus, initializing estimation with the random effects may only be useful if the random-effects specification is kept relatively simple. With more complex random-effects specifications, actual subgroup differences may be more likely captured by the random effects than by the tree structure. The second-lowest MAD was observed for LM trees with clustered covariances (left panel). The combined use of random-effects initialization and cluster-level covariances was not very effective, irrespective of the random-effects specification.

**Table 3:** Variables selected for the first split by each LM tree (top two rows) and LMM tree (bottom eight rows) estimation approach

Random effects	Fitting approach	$u_{1}$	${u}_{2}$	$u_{3}$	$u_{4}$–$u_{25}$	No split
$\hat{\sigma }_{b_0} = \hat{\sigma }_{b_1} = 0$	default	1.000	0.000	0.000	0.000	0.000
$\hat{\sigma }_{b_0} = \hat{\sigma }_{b_1} = 0$	cl.cov.	1.000	0.000	0.000	0.000	0.000
$\hat{\sigma }_{b_0} > 0$, $\hat{\sigma }_{b_1} = 0$	default	1.000	0.000	0.000	0.000	0.000
$\hat{\sigma }_{b_0} > 0$, $\hat{\sigma }_{b_1} = 0$	cl.cov.	1.000	0.000	0.000	0.000	0.000
$\hat{\sigma }_{b_0} > 0$, $\hat{\sigma }_{b_1} = 0$	ran.eff.	0.996	0.002	0.002	0.001	0.000
$\hat{\sigma }_{b_0} > 0$, $\hat{\sigma }_{b_1} = 0$	cl.cov. + ran.eff.	1.000	0.000	0.000	0.000	0.000
$\hat{\sigma }_{b_0} > 0$, $\hat{\sigma }_{b_1} > 0$	default	1.000	0.000	0.000	0.000	0.000
$\hat{\sigma }_{b_0} > 0$, $\hat{\sigma }_{b_1} > 0$	cl.cov.	1.000	0.000	0.000	0.000	0.000
$\hat{\sigma }_{b_0} > 0$, $\hat{\sigma }_{b_1} > 0$	ran.eff.	0.499	0.007	0.006	0.003	0.485
$\hat{\sigma }_{b_0} > 0$, $\hat{\sigma }_{b_1} > 0$	cl.cov. + ran.eff.	1.000	0.000	0.000	0.000	0.000

*Note.* $u_1$ is the true first splitting variable and is binary; all other partitioning variables are continuous, with $u_2$ and $u_3$ being true splitting variables (nodes 2 and 3). $\hat{\sigma }_{b_0}$ and $\hat{\sigma }_{b_1}$ are the estimated standard deviations of the random intercept and slope, respectively

Distributions of the number of splits, separated according to the levels of the data-generating design are presented in Fig. 11 and discussed in Appendix A. The results show a pattern very similar to Fig. 6; no substantial interactions between data-generating and model-specification parameters were observed. Main effects of the data-generating parameters were as expected: Strongest effects were for $\sigma _{b_1}$ and *N*, with higher values resulting in a higher number of splits. The number of noise variables and $\sigma ^2_{b_1}$ had smaller effects, while the correlation between partitioning variables hardly affected the number of implemented splits.

Table 3 shows the variables selected for the first split, and indicates high accuracy for all LM(M) tree-fitting approaches. Only very rarely is $u_1$ not selected for the first split, if a first split was implemented.

## Study II: Comparison with other partitioning methods

Next, we compared the performance of LM(M) trees with that of SEM trees and LongCART. This allowed for evaluating the possible (dis)advantages of global versus local estimation of random-effects parameters, as well as the performance of the different splitting criteria employed by each method. The same data-generating design as in Simulation Study I was employed (Fig. 5). To reduce the number of comparisons, we only included performance of LM(M) trees fitted using clustered covariances, because these showed good performance in Simulation Study I.

### Method

We fitted a total of six SEM trees to every dataset, using two different splitting criteria:

- The default “naive” splitting approach which employs likelihood-ratio tests (LRTs) as the splitting criterion (Brandmaier et al., 2013). That is, for each candidate split, the log-likelihood of the SEM fitted to the observations in the current node is compared against the sum of the log-likelihoods of a two-group SEM, in which the two groups are defined by the candidate split. An LRT can thus be computed for each candidate split, which quantifies the improvement in fit that would result from implementing this split. In each step, the candidate split yielding the highest LRT is selected for splitting, and splitting is continued as long as a candidate split yields a *p* value of the LRT above a pre-specified $\alpha$ level.
- The score-based splitting approach of Arnold et al. (2021). This approach uses the MOB algorithm described in the Introduction, where the parametric model fitted in step (a) is a SEM. While for GLMM trees, parameter stability tests are computed for the fixed-effects parameters only, score-based SEM trees compute parameter stability tests based on both fixed- and random-effects parameters.

For each splitting criterion, three different random-effects specifications were employed:

- None: $\hat{\sigma }_{b_0} = \hat{\sigma }_{b_1} = 0$. Random effects were not estimated and their variances were thus fixed to 0.
- Intercepts: $\hat{\sigma }_{b_0} > 0; \hat{\sigma }_{b_1} = 0$. In every node, the variance of the random intercept was freely estimated; the variance of the random slope was fixed to 0.
- Intercepts and slopes: $\hat{\sigma }_{b_0}> 0; \hat{\sigma }_{b_1} > 0$. In every node, the variances of the random intercept and slope, as well as their correlation, were freely estimated.

To specify the node-specific models for SEM trees, we employed an LGCM specification with the response at each timepoint regressed on a latent intercept and slope. Intercept loadings were fixed to 1; slope loadings were fixed to 0, 1, 2, 3, 4, respectively. Errors were assumed uncorrelated between timepoints and an error variance was freely estimated for each timepoint. We used package *lavaan* (version 0.6-11; Rosseel, 2012) to fit the SEMs and package *semtree* (version 0.9.17; Brandmaier et al., 2013) to fit the SEM trees.

**Fig. 7:**
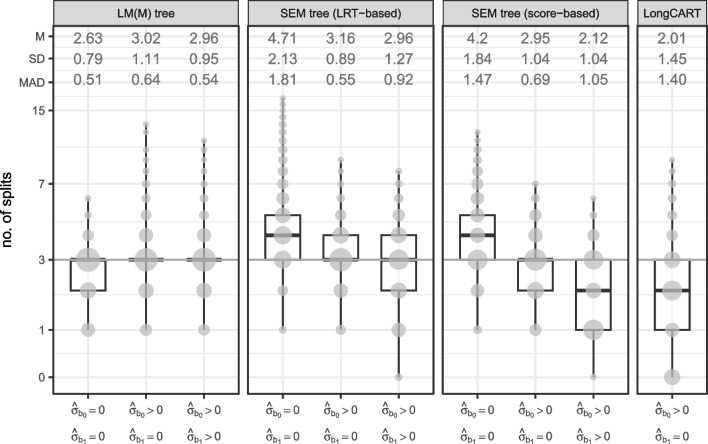
Tree size distributions for LM(M) trees with clustered covariances, SEM trees and LongCART. *Note.* M = mean number of splits; SD = standard deviation of the number of splits; MAD = mean absolute deviation from true tree size. *Gray circles* represent counts, *dark gray horizontal lines* represent true number of splits 3. Distances on the *y*-axis are on the log scale; *x*-axis labels indicate whether variances of the random intercept and slope were fixed to 0, or freely estimated

We fitted a single LongCART tree to each dataset. The LongCART function estimates node-specific models comprising a random intercept term, a default that cannot be overruled. A fixed-effects model was specified with the response regressed on time and a subject-specific random intercept. We used package *LongCART* (version 3.1 Kundu, 2021) to fit these trees.

For all model-fitting parameters not discussed above, default settings were employed. The tree-fitting algorithms employ the same value of $\alpha =0.05$ for evaluating significance of potential splits and deciding whether splitting should continue. Yet, they differ in application of the Bonferroni correction: By default, *semtree* and *LongCART* do not employ a Bonferroni correction for the number of splitting candidates tried at every split, while *glmertree* does. Further, the algorithms have different defaults for the minimum number of observations in a terminal node (argument |minbucket|). In the current experiments, the default setting for *semtree* implements at most 9 ($N=100$) and 24 ($N=250$) splits. Further, *LongCART* and *glmertree* both implement at most 24 ($N=100$) and 61 ($N=250$) splits. To evaluate the effects of these differences, we will compare the number of implemented splits against these maxima.

### Results

#### Tree size

Figure 7 depicts tree size distributions for the different algorithms. SEM trees (middle two panels) performed well, exhibiting overfitting only when no random effects were specified. LRT-based SEM trees tended to implement more splits than score-based SEM trees. Both LRT- and score-based SEM trees yielded lower tree size with more complex random-effect specifications. With the correct random-effects specification (i.e., both random intercept and slope freely estimated), LRT-based SEM trees showed average tree size very close to the true tree size, while under-specification (i.e., only the random intercept freely estimated) increased tree size by 0.2 splits on average. Score-based SEM trees perform best when only random intercepts were estimated, implementing too few splits when random slopes were (correctly) specified. LongCART trees seem to suffer from a lack of power, implementing only two splits on average. Overall, LM(M) trees with clustered covariances showed best performance, but very closely followed by LRT-based SEM trees, which seem more strongly affected by mis-specification of the random effects.

**Table 4:** Variables selected for the first splits by each of the partitioning approaches

Algorithm	Random effects	$u_1$	$u_2$	$u_3$	$u_4$–$u_{25}$	No split
LM(M) tree	$\hat{\sigma }_{b_0} = \hat{\sigma }_{b_1} = 0$	1.000	0.000	0.000	0.000	0.000
(cl.cov.)	$\hat{\sigma }_{b_0} > 0$, $\hat{\sigma }_{b_1} = 0$	1.000	0.000	0.000	0.000	0.000
	$\hat{\sigma }_{b_0} > 0$, $\hat{\sigma }_{b_1} > 0$	1.000	0.000	0.000	0.000	0.000
SEM tree	$\hat{\sigma }_{b_0} = \hat{\sigma }_{b_1} = 0$	1.000	0.000	0.000	0.000	0.000
(LRT-based)	$\hat{\sigma }_{b_0} > 0$, $\hat{\sigma }_{b_1} = 0$	0.998	0.000	0.002	0.000	0.000
	$\hat{\sigma }_{b_0} > 0$, $\hat{\sigma }_{b_1} > 0$	0.992	0.001	0.001	0.002	0.004
SEM tree	$\hat{\sigma }_{b_0} = \hat{\sigma }_{b_1} = 0$	0.761	0.176	0.052	0.012	0.000
(score-based)	$\hat{\sigma }_{b_0} > 0$, $\hat{\sigma }_{b_1} = 0$	0.879	0.077	0.044	0.000	0.000
	$\hat{\sigma }_{b_0} > 0$, $\hat{\sigma }_{b_1} > 0$	0.999	0.000	0.000	0.000	0.001
LongCART	$\hat{\sigma }_{b_0} > 0$, $\hat{\sigma }_{b_1} = 0$	0.000	0.419	0.298	0.092	0.192

*Note.* $u_1$ is the true first splitting variable and is a binary factor; all other partitioning variables are continuous, with $u_2$ and $u_3$ being true splitting variables (nodes 2 and 3). The first column indicates whether the random intercept and/or slope were estimated or not

Distributions of the number of splits, separated according to the levels of the data-generating parameters are depicted and discussed in Appendix A and Fig. 12. They are omitted here, as they show a pattern very similar to Fig. 7. Of the four data-generating parameters, *N* and $\sigma _{b_0}^2$ showed the strongest effects, with higher values resulting in a higher number of splits, as expected. The number of splits implemented by SEM trees was most strongly affected by the data-generating parameters when the random effects were mis-specified (i.e., random intercepts and/or slope fixed to 0). None of the algorithms reached the maximum number of splits retained as specified by the |minbucket| argument in any of the simulated datasets, suggesting that this parameter does not affect tree size. The strong effect of sample size suggests the significance level $\alpha$ for continuation of splitting is much more important. With $N=100$, underfitting is more likely, while with $N=250$, overfitting is more likely, suggesting that the default $\alpha$ performs well in these simulations. Combined with the very small effect of the number of potential partitioning variables, the default (lack of) Bonferroni correction also performs well.

#### Split selection

Table 4 presents variable selection frequencies for the first split in the fitted trees. LRT-based SEM trees provide almost perfect accuracy for the first split. Score-based SEM trees provide near-perfect accuracy when random effects were correctly specified. When random effects were mis-specified, score-based SEM trees selected the wrong variable for the first split in about 12% of datasets. Closer inspection of stability tests for individual models and parameters suggested that the score-based tests for SEMs are more sensitive to instability in the fixed slope than in the fixed intercept, explaining why $u_2$ or $u_3$ were often selected for the first split.

**Fig. 8:**
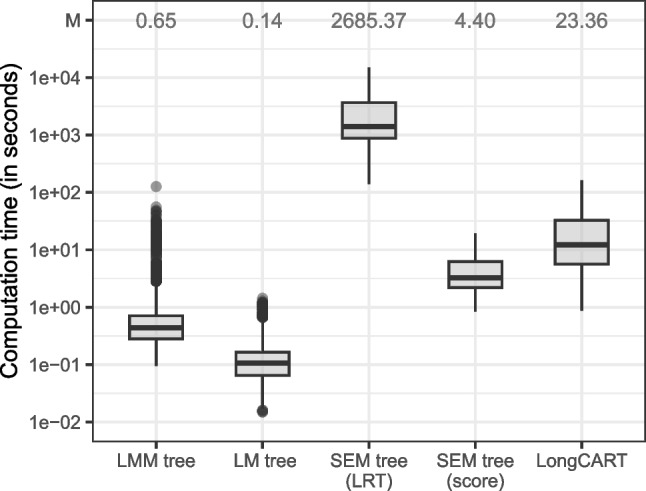
Computation time distributions for the different partitioning methods. *y*-axis is on the log scale; M = mean computation time in seconds

LongCART trees exhibit low accuracy for recovering the first split, selecting the wrong variable in all datasets where at least one split was implemented. LongCART showed a strong tendency to select $u_2$ or $u_3$ for the first split. Closer inspection of the fitted LongCART trees revealed that in 99% percent of datasets in which no splits were implemented, $u_1$ was found to be the strongest splitting candidate, but the parameter stability tests did not reach significance. This suggests the tests proposed by Kundu & Harezlak (2019) are less sensitive to instability of the intercept (compared to slope instability), or less sensitive to instability with respect to categorical covariates (compared to continuous covariates).

#### Computation time

Figure 8 presents computation time distributions for the partitioning algorithms. A clear computational advantage is observed for LM trees. LMM trees require somewhat longer computation times because of the estimation of random effects. Yet, this increase seems minor compared to the computation times required by LongCART and score-based SEM trees. As expected, LRT-based SEM trees require strikingly long computation times.

## Study III: Partitioning academic trajectories

### Method

#### Dataset

We analyzed trajectories of children’s reading, math and science abilities from the Early Childhood Longitudinal Study-Kindergarten class of 1998–1999 (ECLS-K; National Center for Education Statistics, 2010). Data were collected from 21,304 children from 1,018 schools across the USA. Assessments took place from kindergarten in 1998 through 8th grade in 2007, here we focus on assessments from kindergarten, 1st, 3rd, 5th and 8th grade.

Response variables are reading, math, and science abilities, which were assessed using multi-item cognitive tests. The scores on the tests represent latent ability estimates computed from an IRT model. Reading and math abilities were assessed in all five rounds of data collection, science knowledge was not assessed kindergarten and 1st grade, only in 3rd, 5th and 8th grade. We analyzed data from children who completed all assessments yielding $N = 6277$ for reading; $N = 6512$ for math; $N = 6625$ for science. Figure 13 (Appendix B) shows the distribution of reading, math and science ability scores separated by grade, which reveals approximately symmetric distributions, with lower kurtosis than a normal distribution.

Time was measured as the number of months since the baseline assessment. In order to obtain approximately linear trajectories, we transformed the timing metric based on visual inspection of the observed trajectories: $\textrm{months}^{1/2}$ was used for reading and math trajectories, and $\textrm{months}^{2/3}$ for science trajectories, which showed less non-linearity and therefore required a power closer to one. Figure 14 (Appendix B) shows the trajectories before and after transformation. We chose a fractional exponent over the quadratic polynomials sometimes recommended in the literature to account for growth slowing down over time (e.g., Grimm et al., 2011), as it resulted in a lower number of parameters to be estimated.

We used 11 time-invariant covariates as potential partitioning variables, all assessed at baseline: gender (51.1% male); age in months (range 53–96; M = 6.14 years); race (eight categories); first time in kindergarten (yes/no); socio-economic status (range −5 to 3); fine motor skills (e.g., drawing figures; range 0–9); gross motor skills (e.g., ability to hop, skip and jump; range 0–8); interpersonal skills (range 1–4); self-control (range 1–4); internalizing problem behavior (range 1–4); externalizing problem behavior (range 1–4).

#### Fitting approaches

We applied five LM(M) trees to the data, focusing on the original GLMM tree approach and those that performed well in the simulations:

- An LM tree ($\hat{\sigma }_{b_0} = \hat{\sigma }_{b_1} = 0$) using clustered covariances in the parameter-stability tests.
- An LMM tree using observation-level covariances, with a random intercept freely estimated ($\hat{\sigma }_{b_0} > 0$, $\hat{\sigma }_{b_1} = 0$) and initialization assuming zero random effects.
- An LMM tree using clustered covariances, with a random intercept freely estimated ($\hat{\sigma }_{b_0} > 0$, $\hat{\sigma }_{b_1} = 0$) and initialization assuming zero random effects.
- An LMM tree using clustered covariances, with both random intercept and slope freely estimated ($\hat{\sigma }_{b_0} > 0$, $\hat{\sigma }_{b_1} \!> 0$) and initialization assuming zero random effects.
- An LMM tree using observation-level covariances, with a random intercept freely estimated ($\hat{\sigma }_{b_0} > 0$, $\hat{\sigma }_{b_1} = 0$) and random-effects initialization with the full-sample estimate.

Although LRT- and score-based SEM trees performed very well in the simulations, they could not be used in this study because growth-curve SEMs do not allow for incorporating continuous time. For comparison, we therefore fitted two linear mixed models, both with random intercept and slope of time freely estimated ($\hat{\sigma }_{b_0} > 0$, $\hat{\sigma }_{b_1} > 0$):

- A simple LMM, with a linear fixed effect of time, reflecting a situation where no subgroups are detected or used for prediction.
- A complex LMM, with a linear fixed effect of time, as well as main effects and interactions with time of all possible partitioning variables.

**Table 5:** Cross-validated mean squared errors for each of the response variables

	Math			Reading			Science		
	*M*	*SD*	$R^{2}$	*M*	*SD*	$R^{2}$	*M*	*SD*	$R^{2}$
LM tree$^c$	0.1408	0.004	0.806	0.1270	0.003	0.806	0.4204	0.013	0.530
LMM tree$^i$	0.1473	0.006	0.797	0.1315	0.005	0.799	0.4155	0.015	0.535
LMM tree$^{i,c}$	0.1401	0.005	0.807	0.1266	0.003	0.807	0.4197	0.014	0.530
LMM tree$^{i,s,c}$	0.1407	0.005	0.806	0.1267	0.003	0.807	0.4189	0.014	0.531
LMM tree$^{i,r}$	0.1473	0.006	0.797	0.1315	0.005	0.799	0.4129	0.012	0.538
LMM$^t$	0.1646	0.001	0.773	0.1409	0.001	0.785	0.5031	0.002	0.437
LMM$^a$	0.1159	0.003	0.840	0.1124	0.002	0.828	0.3506	0.008	0.608

*Note.* Means and standard deviations computed over 100 cross-validation repetitions. $R^2$ was computed as $1-\frac{\text {mean(MSE)}}{\text {var}(y)}$. $^c$ cluster-level covariances; $^r$ estimation initialized with random effects; $^i$ random-intercept variance freely estimated; $^s$ random-slope variance freely estimated; $^t$ LMM with fixed effect of time; $^a$ LMM with fixed effects of time, all covariates and all time-by-covariate interactions

#### Evaluation of performance

The ECLS-K datasets have exceptionally large sample sizes, so we employed random sampling to obtain training samples of $N=250$ children, likely more representative of real-world studies in psychology. We performed 100 repetitions for each response variable (math, reading, or science). We evaluated predictive accuracy by computing the mean squared difference between predicted and observed response variable values (MSE) for all children not included in the training sample in the current repetition. Such separation of train and test observations does not allow for using random effects in computing predictions; cross-validated MSEs thus only quantify accuracy of the fixed-effects parameters. Model complexity was measured by two indicators: Tree size as quantified by the number of splits in each tree, and the number of covariates used in the final model. Pairwise *t*-tests with Bonferroni correction to account for multiple testing were applied to the MSEs of all methods to evaluate statistical significance of differences.

### Results

**Fig. 9:**
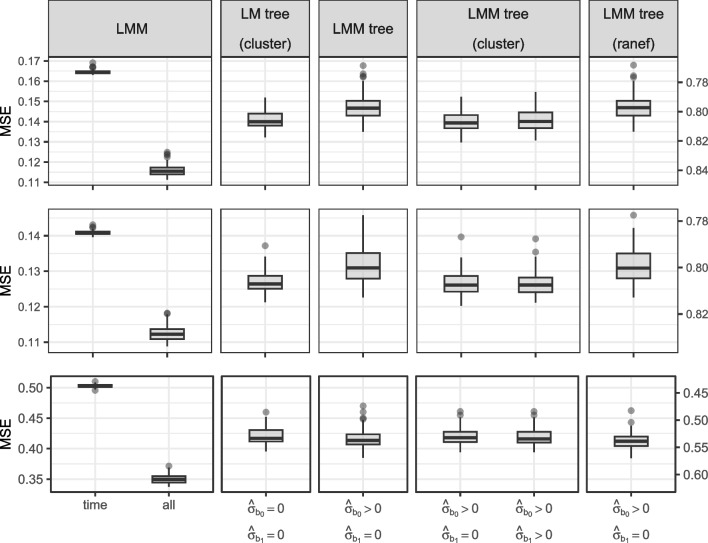
Mean squared errors for trees fitted to math (*top row*), reading (*middle row*) and science (*bottom row*) ability trajectories. Secondary *y*-axis on the right quantifies the proportion of variance explained, computed as $1 - \frac{\text {mean(MSE)}}{\text {var}(y)}$

Table 5 and Fig. 9 present MSE and $R^2$ distributions. Simple LMMs comprising only a fixed effect of time performed worst, but their $R^2$ values ranging from 0.43 to 0.79 illustrate that time is the most important predictor of math, reading and science abilities. The complex LMMs, comprising fixed effects of time and all covariates as well as their interactions, showed best performance, with $R^2$ values ranging from 0.58 to 0.85. Table 6, which shows the number of covariates used for prediction, indicates this performance gain comes at a considerable cost in complexity.

Predictive performance of LM(M) trees strikes a balance between the simple and complex LMMs. As shown in Table 5, LM(M) trees with clustered covariances provide predictive accuracy closer to the complex than the simple LMMs, while using only a fraction of the covariates: 39% of covariates for predicting math, 28% of covariates for reading, and 27% of covariates for science trajectories, on average. As such, LMM trees substantially improve upon the simple, no-subgroup LMMs using only a few partitioning variables.

Between the different LMM-tree-fitting approaches, differences in performance are small, all $R^2$ differences $< 0.01$. There were no significant differences in performance between default LMM trees and LMM trees with random-effects initialization for any of the three outcomes. In contrast, the three LM(M) trees using clustered covariances performed significantly better for the math and reading outcomes. For the science outcomes, no significant differences between LM(M) trees were observed. Thus, cluster-level covariances seem to provide the best predictive performance for LM(M) trees.

**Fig. 10:**
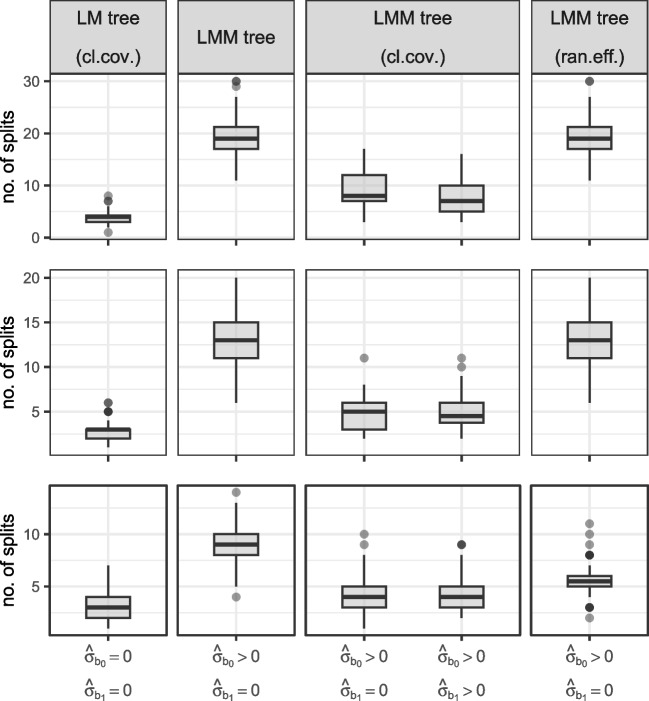
Sizes of trees fitted to math (*top row*), reading (*middle row*), and science (*bottom row*) ability trajectories

**Fig. 11:**
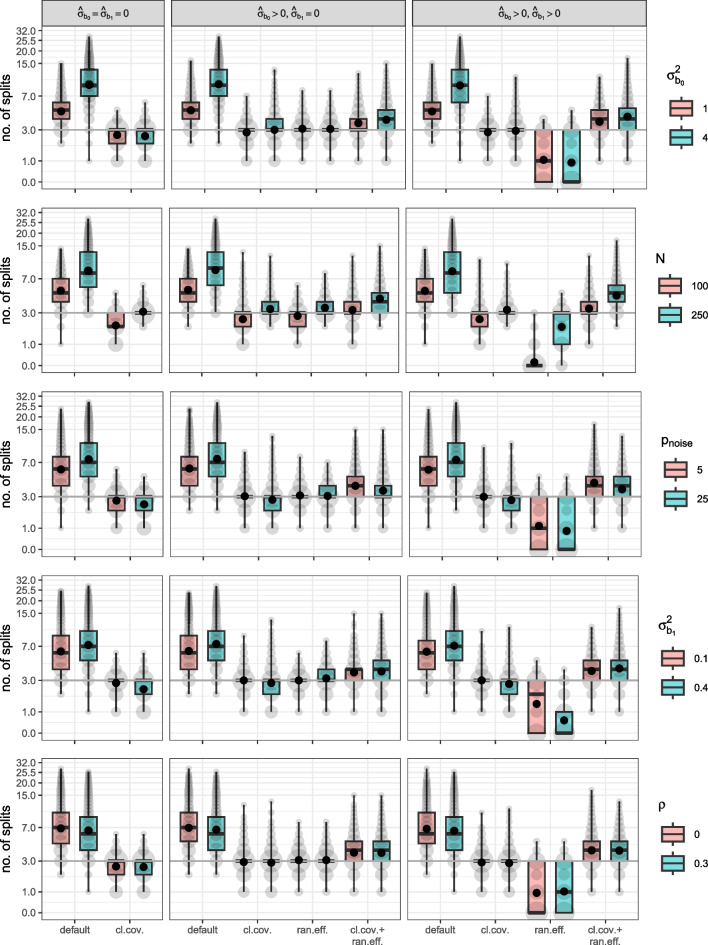
Effects of data-generating parameters on tree size for LM(M) trees. *Note.* *Black dots* represent means, *gray circles * represent counts, *dark gray horizontal lines* represent true number of splits 3. Distances on *y*-axis are on a log scale. $\sigma _{b_0}^2$ = variance of random intercept; $\sigma _{b_1}^2$ = variance of random slope; *N* = sample size at level 2; $p_{noise}$ = number of noise variables; $\rho$ = correlation between partitioning variables

**Fig. 12:**
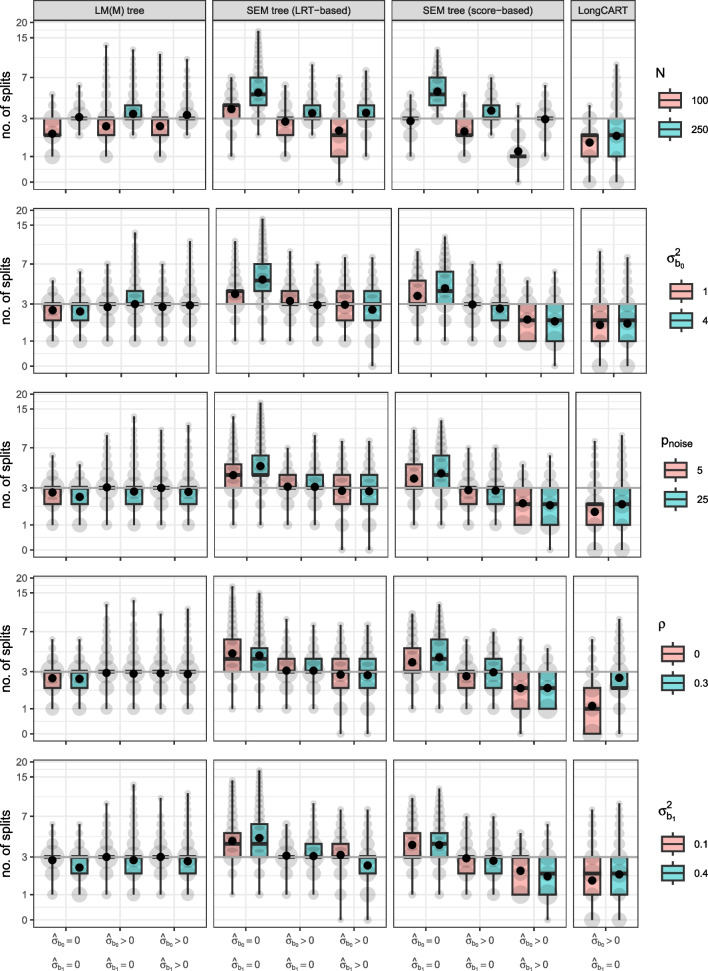
Effect of data-generating parameters on tree size for LM(M), SEM and LongCART trees. *Note.* *Black dots *represent means, *gray circles* represent counts, *dark gray horizontal lines* represent true number of splits 3. Distances on *y*-axis are on a log scale. *N* = sample size at level 2; $\sigma _{b_0}^2$ = variance of random intercept; $\sigma _{b_1}^2$ = variance of random slope; $p_{noise}$ = number of noise variables; $\rho$ = correlation between partitioning variables

**Fig. 13:**
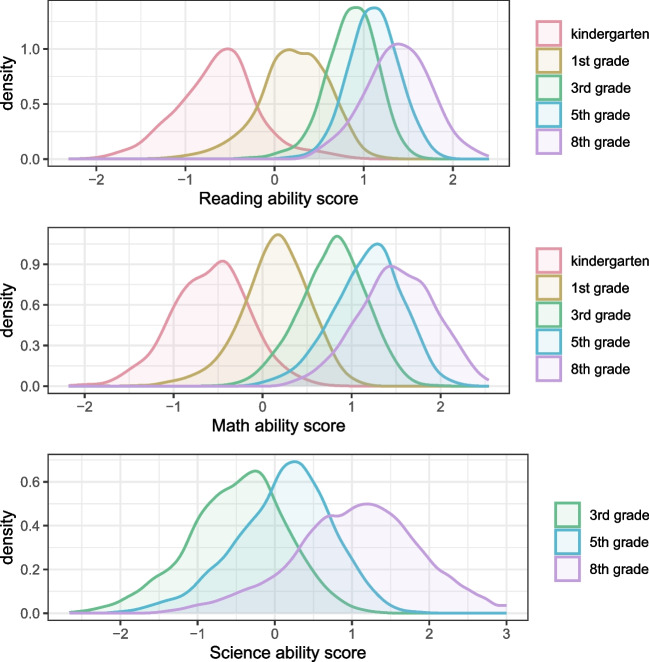
Density plots of reading, math and science ability scores, separated by grade

**Fig. 14:**
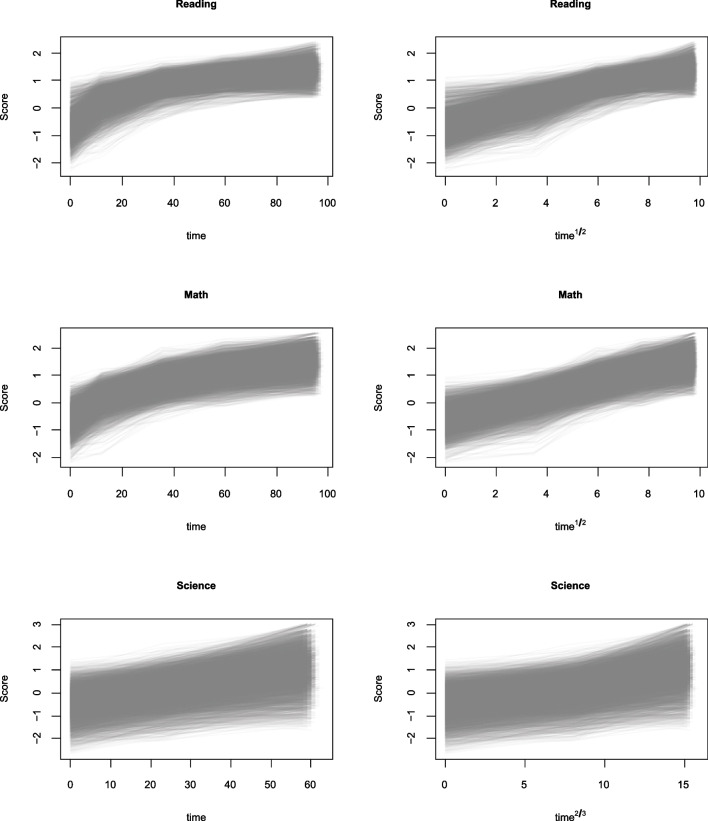
Observed trajectories before (*left column*) and after (*right column*) transforming time for reading ($N = 6277$), math ($N = 6512$) and science ($N = 6625$) abilities in the application. *Note.* Before transformation, time is measured in months after baseline assessment

Figure 10 presents tree size distributions for the LM(M) trees. Similar to the simulation study’s results, default LMM trees implement the largest number of splits. Cluster-level covariances provide a robust reduction in the number of splits. Given their non-distinguishable predictive performance, LM trees with clustered covariances may be preferred if obtaining a sparse result is critical. LMM trees with clustered covariances may provide less sparsity but better predictive accuracy, irrespective of whether random intercepts and/or slopes were estimated. In contrast to the simulation study results, random-effects initialization resulted in tree sizes similar to the default fitting approach in these experiments.

## Discussion

The simulations showed that the proposed extensions of GLMM trees are effective for partitioning LGCMs. Use of clustered covariances seems most effective and their good performance was largely unaffected by (mis-)specification of the random effects. Clustered covariances may therefore be the optimal choice in most practical applications. Initializing estimation with the random effects was also effective, but only when the random-effects specification is kept simple (i.e., no estimation of random slopes). Combining cluster-level covariances and random-effects initialization worsened performance and is thus not recommended.

**Table 6:** Mean number of covariates used in the final models

	Math		Reading		Science	
	M	SD	M	SD	M	SD
LM tree$^c$	3.01	0.81	2.36	0.86	2.65	0.93
LMM tree$^i$	8.25	1.16	7.03	1.31	5.62	1.19
LMM tree$^{i,c}$	5.15	1.37	3.36	1.26	3.19	1.28
LMM tree$^{i,r}$	8.22	1.14	7.04	1.33	4.01	1.07
LMM tree$^{i,s,c}$	4.65	1.58	3.42	1.23	3.22	1.25
LMM$^{t}$	0.00	0.00	0.00	0.00	0.00	0.00
LMM$^{a}$	11.00	0.00	11.00	0.00	11.00	0.00

*Note.* Means and standard deviations computed over 100 cross-validation repetitions. $^c$ cluster-level covariances; $^r$ estimation initialized with random effects; $^i$ random-intercept variance freely estimated; $^s$ random-slope variance freely estimated; $^t$ LMM with fixed effect of time; $^a$ LMM with fixed effects of time, all covariates and all time-by-covariate interactions

Strong performance of clustered covariances was also observed in partitioning real-world academic trajectories. They provided substantially smaller trees for all outcomes and better or equal predictive accuracy. In comparison to cluster-level covariances, random-effects initialization resulted in larger trees for all outcomes and worse performance for the reading and math outcomes. GLMM trees provided a substantial boost in predictive performance compared to simple global LGCMs.

Yet, GLMM trees were outperformed by LGCMs comprising main effects and interactions with time of all baseline covariates. This suggests that the underlying assumption of these LGCMs – that all covariates and their interaction with time were relevant for predictions – was correct. GLMM trees did not have this prior knowledge and needed to learn the interactions from samples of limited size ($N=250$). With larger samples, the complexity of the tree may increase to capture more interactions. At the same time, GLMM trees employing clustered covariances selected only one-third of baseline covariates to distinguish subgroups, thus providing simpler and easier to interpret results. This is in line with earlier research showing that trees may not provide state-of-the-art accuracy but do provide a very good trade-off between accuracy and complexity (Hand, 2006; Fokkema et al., 2022). Especially if there are a large number of possibly relevant covariates, or categorical covariates with many levels, GLMM trees may provide an interpretable alternative to LGCMs comprising all covariates.

The simulations showed comparable performance of LM(M) and SEM trees in partitioning LGCMs. SEM trees may however be more sensitive to mis-specification of the random effects, with under-specification resulting in too many splits. In line with results of Arnold et al. (2021), we found score-based SEM trees to have somewhat lower power than LRT-based SEM trees, but at a much lower computational cost. LongCART trees often selected the wrong partitioning variable for the first split, and were outperformed by LM(M) and SEM trees. The LongCART parameter stability tests (Kundu & Harezlak, 2019) may be underpowered for detecting instability of the fixed intercept, or for detecting instability with respect to categorical covariates.

The simulations clearly illustrated the lower computational burden of GLMM trees. This is in large part due to their local-global estimation approach, where fixed-effects parameters are estimated locally within a node and random-effects parameters are estimated globally, using all observations. In contrast, SEM trees and LongCART fit the full mixed-effects model in each node, which substantially increases computational load. The local-global estimation approach also reduces model complexity, because a lower number of random-effects parameters need to be estimated.

Yet, a possible downside of the local-global estimation approach is that it does not allow for recovering subgroups with differences in random-effects parameters. When there is a specific interest in subgroups with different random-effects parameters, score-based SEM trees should likely be preferred. Alternatively, researchers may want to use the parameter stability tests for mixed-effects models developed by Wang & Merkle (2018) (see also Wang et al., 2021, 2022). These will be useful, for example, when the number of or distances between timepoints differ between respondents so SEM-based growth curve models cannot be applied (McNeish & Matta, 2018).

The current evaluations were limited to Gaussian responses and LGCMs. Future studies should assess performance of GLMM trees in partitioning longitudinal data with, for example, binomial or count responses. We expect that the strong performance of cluster-level covariances generalizes to other settings where partitioning covariates are measured at higher levels, in longitudinal as well as in otherwise nested or clustered data structures; but this remains to be evaluated empirically. Finally, we used the outer-product-of-gradients (OPG) estimator for computing (clustered) covariances. Though computationally more burdensome, future work could assess potential benefits of using the full sandwich estimator.

## Data Availability

All methods used are implemented in R and associated packages, as described in the Method section.

## Effects of data-generating parameters on tree size

For LM(M) trees (Fig. 11), use of cluster-level covariances provided the most robust improvement in split recovery. For the data-generating parameters, the effects on tree size were strongest for $\sigma _{b_0}$, followed by *N*, *p*, $\sigma _{b_1}$ and $\rho$. Higher values of $\sigma _{b_0}$, *N*, *p* and $\sigma _{b_1}$ tend to yield higher numbers of splits, while the effect of $\rho$ is minimal.

For SEM trees and LongCART (Fig. 12), strongest effects were observed for *N*, followed by $\sigma _{b_0}$, $p_{noise}$, $\rho$ and $\sigma _{b_1}$. Results for *N* were as expected for all methods: More splits are implemented with higher sample size. Both LRT- and score-based SEM trees seem only affected by levels of $\sigma _{b_0}$, $p_{noise}$ and $\rho$ under misspecification of the random effects. Especially when both random intercept and slope variances are fixed to 0, higher levels of $\sigma _{b_0}$ and $p_{noise}$ yield more splits with both SEM tree approached. LRT-based SEM trees seem unaffected by levels of $\rho$, while score-based SEM trees seem to implement a larger number of splits with increasing values of $\rho$. This pattern seems to be reversed for increased magnitude of $\sigma _{b_1}$, which yields a lower number of splits for both SEM tree approaches, but only when the random effects are correctly specified. LongCART implemented more splits with higher levels of $\rho$, $\sigma _{b_1}$, *N*, $p_{noise}$ and $\sigma _{b_0}$.

## Footnotes

1 Further details on the source of these data and the scaling of time are provided in Study III.

2 Both IT methods specifically target subgroups with different time-by-treatment interactions, so are not generally applicable for partitioning growth curve models.

3 Although GLMM trees are not restricted to having the same set of timepoints for each subject, this design allowed for comparison with SEM trees in Study II.

## Code availability

Data and R code are available as supplementary materials. None of the experiments were preregistered.

## References

[CR1] Abdolell, M., LeBlanc, M., Stephens, D., & Harrison, R. V. (2002). Binary partitioning for continuous longitudinal data: Categorizing a prognostic variable. *Statistics in Medicine,**21*(22), 3395–3409. 10.1002/sim.1266 10.1002/sim.1266 12407680

[CR2] Arnold, M., Voelkle, M.C., & Brandmaier, A. M. (2021). Score-guided structural equation model trees. *Frontiers in Psychology,**11*, 3913. 10.3389/fpsyg.2020.564403 10.3389/fpsyg.2020.564403 PMC7875879 33584404

[CR3] Athey, S., & Imbens, G. (2016). Recursive partitioning for heterogeneous causal effects. *Proceedings of the National Academy of Sciences,**113*(27), 7353–7360. 10.1073/pnas.1510489113 10.1073/pnas.1510489113 PMC4941430 27382149

[CR4] Bates, D., Mächler, M., Bolker, B., & Walker, S. (2015). Fitting linear mixed-effects models using lme4, *Journal of Statistical Software,**67*(1), 1–48. 10.18637/jss.v067.i01

[CR5] Brandmaier, A. M., von Oertzen, T., McArdle, J. J., & Lindenberger, U. (2013). Structural equation model trees. *Psychological Methods,**18*(1), 71. 10.1037/a0030001 10.1037/a0030001 PMC4386908 22984789

[CR6] Brandmaier, A. M., Von Oertzen, T., Ghisletta, P., Lindenberger, U., & Hertzog, C. (2018). Precision, reliability, and effect size of slope variance in latent growth curve models: Implications for statistical power analysis. *Frontiers in Psychology,**9*, 294. 10.3389/fpsyg.2018.00294 29755377 10.3389/fpsyg.2018.00294 PMC5932409

[CR7] Eo, S. H., & Cho, H. (2014). Tree-structured mixed-effects regression modeling for longitudinal data. *Journal of Computational and Graphical Statistics,**23*(3), 740–760. 10.1080/10618600.2013.794732

[CR8] Fokkema, M., Iliescu, D., Greiff, S., & Ziegler, M. (2022). Machine learning and prediction in psychological assessment: Some promises and pitfalls. *European Journal of Psychological Assessment,**38*(3), 165–175. 10.1027/1015-5759/a000714

[CR9] Fokkema, M., Smits, N., Zeileis, A., Hothorn, T., & Kelderman, H. (2018). Detecting treatment-subgroup interactions in clustered data with generalized linear mixed-effects model trees. *Behavior Research Methods,**50*(5), 2016–2034. 10.3758/s13428-017-0971-x 10.3758/s13428-017-0971-x 29071652

[CR10] Fu, W., & Simonoff, J. S. (2015). Unbiased regression trees for longitudinal and clustered data. *Computational Statistics & Data Analysis,**88*, 53–74. 10.1016/j.csda.2015.02.004

[CR11] Grimm, K. J., Ram, N., & Hamagami, F. (2011). Nonlinear growth curves in developmental research. *Child Development,**82*(5), 1357–1371. 10.1111/j.1467-8624.2011.01630.x 21824131 10.1111/j.1467-8624.2011.01630.x PMC3169758

[CR12] Hajjem, A., Bellavance, F., & Larocque, D. (2011). Mixed effects regression trees for clustered data. *Statistics & Probability Letters,**81*(4), 451–459. 10.1016/j.spl.2010.12.003

[CR13] Hajjem, A., Bellavance, F., & Larocque, D. (2014). Mixed-effects random forest for clustered data. *Journal of Statistical Computation and Simulation,**84*(6), 1313–1328. 10.1080/00949655.2012.741599

[CR14] Hajjem, A., Larocque, D., & Bellavance, F. (2017). Generalized mixed effects regression trees. *Statistics & Probability Letters,**126*, 114–118. 10.1016/j.spl.2017.02.033

[CR15] Hand, D. J. (2006). Classifier technology and the illusion of progress. *Statistical Science,**21*(1), 1–15. 10.1214/088342306000000060

[CR16] Hansen, B. E. (1997). Approximate asymptotic values for structural-change tests. *Journal of Business & Economic Statistics,**15*(1), 60–67. 10.2307/1392074

[CR17] Hothorn, T., & Zeileis, A. (2015). partykit: A modular toolkit for recursive partytioning in R. *Journal of Machine Learning Research,**16*, 3905–3909.

[CR18] Kundu, M. G. (2021). LongCART: Recursive partitioning for longitudinal data and right censored data using baseline covariates [Computer software manual]. Retrieved from https://CRAN.R-project.org/package=LongCART

[CR19] Kundu, M. G., & Harezlak, J. (2019). Regression trees for longitudinal data with baseline covariates. *Biostatistics & Epidemiology,**3*(1), 1–22. 10.1080/24709360.2018.1557797 10.1080/24709360.2018.1557797 PMC6347409 30693349

[CR20] Lee, S. K. (2005). On generalized multivariate decision tree by using GEE. *Computational Statistics & Data Analysis,**49*(4), 1105–1119. 10.1016/j.csda.2004.07.003

[CR21] Loh, W. Y. (2002). Regression trees with unbiased variable selection and interaction detection. *Statistica Sinica,**12*(2), 361–386.

[CR22] McNeish, D., & Matta, T. (2018). Differentiating between mixed-effects and latent-curve approaches to growth modeling. *Behavior Research Methods,**50*(4), 1398–1414. 10.3758/s13428-017-0976-5 10.3758/s13428-017-0976-5 29067672

[CR23] Merkle, E. C., Fan, J., & Zeileis, A. (2014). Testing for measurement invariance with respect to an ordinal variable. *Psychometrika,**79*(4), 569–584. 10.1007/s11336-013-9376-7 10.1007/s11336-013-9376-7 24282129

[CR24] National Center for Education Statistics (2010). Early childhood longitudinal study program: Kindergarten class of 1998–1999 (ECLS-K), Retrieved from https://nces.ed.gov/ecls/kindergarten.asp

[CR25] Pinheiro, J. C., & Bates, D. M. (2000). *Mixed-effects models in S and S-PLUS*. New York: Springer-Verlag. 10.1007/b98882

[CR26] R Core Team (2022). R: A language and environment for statistical computing [computer software manual]. Vienna, Austria. Retrieved from https://www.R-project.org/

[CR27] Rosseel, Y. (2012). lavaan: An R package for structural equation modeling. *Journal of Statistical Software,**48*(2),1–36. 10.18637/jss.v048.i02

[CR28] Sela, R. J., & Simonoff, J. S. (2012). RE-EM trees: A data mining approach for longitudinal and clustered data. *Machine Learning,**86*(2), 169–207. 10.1007/s10994-011-5258-3

[CR29] Shih, Y. S. (2004). A note on split selection bias in classification trees. *Computational Statistics & Data Analysis,**45*(3), 457–466. 10.1016/s0167-9473(03)00064-1

[CR30] Shih, Y. S., & Tsai, H. W. (2004). Variable selection bias in regression trees with constant fits. *Computational Statistics & Data Analysis,**45*(3), 595–607. 10.1016/s0167-9473(03)00036-7

[CR31] Stegmann, G., Jacobucci, R., Serang, S., & Grimm, K. J. (2018). Recursive partitioning with nonlinear models of change. *Multivariate Behavioral Research,**53*(4), 559–570. 10.1080/00273171.2018.1461602 10.1080/00273171.2018.1461602 29683722

[CR32] Su, X., Meneses, K., McNees, P., & Johnson, W. O. (2011). Interaction trees: Exploring the differential effects of an intervention programme for breast cancer survivors. *Journal of the Royal Statistical Society C,**60*(3), 457–474. 10.1111/j.1467-9876.2010.00754.x

[CR33] Wang, T., Graves, B., Rosseel, Y., & Merkle, E. C. (2022). Computation and application of generalized linear mixed model derivatives using lme4. *Psychometrika,**87*(3), 1173–1193. 10.1007/s11336-022-09840-2 10.1007/s11336-022-09840-2 35118605

[CR34] Wang, T., & Merkle, E. C. (2018). merDeriv: Derivative computations for linear mixed effects models with application to robust standard errors. *Journal of Statistical Software, Code Snippets,**87*(1), 1–16. 10.18637/jss.v087.c01

[CR35] Wang, T., Merkle, E. C., Anguera, J. A., & Turner, B. M. (2021). Score-based tests for detecting heterogeneity in linear mixed models. *Behavior Research Methods,**53*(1), 216–231. 10.3758/s13428-020-01375-7 10.3758/s13428-020-01375-7 32666394

[CR36] Wei, Y., Liu, L., Su, X., Zhao, L., & Jiang, H. (2020). Precision medicine: Subgroup identification in longitudinal trajectories. *Statistical Methods in Medical Research*, *29*(9). 10.1177/0962280220904114 10.1177/0962280220904114 PMC8357634 32070237

[CR37] Zeileis, A., & Hornik, K. (2007). Generalized m-fluctuation tests for parameter instability. *Statistica Neerlandica,**61*(4), 488–508. 10.1111/j.1467-9574.2007.00371.x

[CR38] Zeileis, A., Hothorn, T., & Hornik, K. (2008). Model-based recursive partitioning. *Journal of Computational and Graphical Statistics,**17*(2), 492–514. 10.1198/106186008x319331

[CR39] Zeileis, A., Köll, S., Graham, N. (2020). Various versatile variances: An object-oriented implementation of clustered covariances in R. *Journal of Statistical Software,**95*(1). 10.18637/jss.v095.i01

